# Multidrug-Resistant Bacteria Isolated from Surface Water in Bassaseachic Falls National Park, Mexico

**DOI:** 10.3390/ijerph13060597

**Published:** 2016-06-16

**Authors:** Ma. Carmen E. Delgado-Gardea, Patricia Tamez-Guerra, Ricardo Gomez-Flores, Francisco Javier Zavala-Díaz de la Serna, Gilberto Eroza-de la Vega, Guadalupe Virginia Nevárez-Moorillón, María Concepción Pérez-Recoder, Blanca Sánchez-Ramírez, María del Carmen González-Horta, Rocío Infante-Ramírez

**Affiliations:** 1 Departamento de Microbiología e Inmunología, Facultad de Ciencias Biológicas, Universidad Autónoma de Nuevo León Ave. Universidad s/n, San Nicolás de los Garza 66450, N.L., Mexico; carmen_060@hotmail.com (M.C.E.D.-G.); patamez@hotmail.com (P.T.-G.); rgomez60@hotmail.com (R.G.-F.); 2 Laboratorio de Biotecnología, Facultad de Ciencias Químicas, Universidad Autónoma de Chihuahua, Circuito Nuevo Campus Universitario s/n, Chihuahua 31125, Mexico; fzavala@uach.mx (F.J.Z.-D.S.); gerosa@uach.mx (G.E.-V.); vnevare@uach.mx (G.V.N.-M.); bsanche@uach.mx (B.S.-R.); carmengonzalez@uach (M.C.G.-H.); 3 Comisión Nacional de Áreas Naturales Protegidas, Dirección Regional Norte y Sierra Madre Occidental, Parque Nacional Cascada de Bassaseachic, Ocampo, Chihuahua 31203, Mexico; crecoder@conanp.gob.mx

**Keywords:** water, pollution, antibiotic, multidrug resistance, enteric, microorganisms, MPN, environment

## Abstract

Bacterial pathogens are a leading cause of waterborne disease, and may result in gastrointestinal outbreaks worldwide. Inhabitants of the Bassaseachic Falls National Park in Chihuahua, Mexico show seasonal gastroenteritis problems. This aim of this study was to detect enteropathogenic microorganisms responsible for diarrheal outbreaks in this area. In 2013, 49 surface water samples from 13 selected sampling sites along the Basaseachi waterfall and its main rivers, were collected during the spring, summer, autumn, and winter seasons. Fecal and total coliform counts were determined using standard methods; the AutoScan-4 system was used for identification of isolates and the antibiotic resistance profile by challenging each organism using 21 antibiotics. Significant differences among seasons were detected, where autumn samples resulted in the highest total (*p* < 0.05) and fecal (*p* < 0.001) coliform counts, whereas the lowest total coliform counts were recorded in spring. Significant differences between sampling sites were observed, where samples from sites 6, 8, and 11 had the highest total coliform counts (*p* < 0.009), whereas samples from site 9 exhibited the lowest one. From the microbiological analysis, 33 bacterial isolates from 13 different sites and four sampling seasons were selected; 53% of isolates were resistant to at least one antibiotic, and 15% exhibited a multidrug resistance (MDB) phenotype. MDB were identified as *Klebsiella oxytoca* (two out of four identified isolates), *Escherichia coli* (2/7), and *Enterobacter cloacae* (1/3). In addition, some water-borne microorganisms exhibited resistance to cefazoline, cefuroxime, ampicillin, and ampicillin-sulbactam. The presence of these microorganisms near rural settlements suggests that wastewater is the contamination source, providing one possible transmission mechanism for diarrheal outbreaks.

## 1. Introduction

Bacteria are one of the most important pathogens in waterborne diseases, and these organisms cause gastrointestinal outbreaks worldwide. Many human diseases are transmitted as a result of the ingestion of contaminated water and food. There are nearly 1.7 billion cases of diarrhea per year worldwide; this condition is the second leading cause of death in children less than five years old [1,2,3] and kills nearly 760,000 children per year. These health problems are common in rural areas in developing countries, which lack access to safe, fresh drinking water, and they remain a serious problem [4,5]. Enteric bacteria are present in the human gastrointestinal system and feces of all warm-blooded animals. These bacteria are commonly dumped into water environments through the runoff from farms and rural settlements with industrial and agricultural pollutants, lacking of wastewater treatment systems [1,6].

Bacterial pathogens potentially transmitted through water ingestion include *Escherichia coli*, *Klebsiella* sp*., Salmonella* *enterica* subsp. *Enterica* Serovar Typhi*, Salmonella* *enterica* subsp. *Enterica* Serovar Paratyphi*, Shigella* spp., *Vibrio cholerae*, *Acinetobacter* sp., *Clostridium* spp., and *Bacillus anthracis*. Other bacterial pathogens that can survive in soil and water include *Legionella*, *Burkholderia pseudomallei* (also known as *Pseudomonas pseudomallei*), and atypical mycobacteria [1,2,5]. Since 1895, *E. coli* has been identified as an indicator of fecal pollution in water samples; not all species of *E. coli* are pathogenic, but a few of these bacteria, such as *E. coli* O157:H7, can be fatal [7,8]. The most probable number technique is commonly used as an index to determine the microbial safety of drinking water; this method is performed in multiple tubes showing the probabilistic value of the bacteria in 100 mL of water, as described in ISO 6461 [9].

Untreated waste introduces additional resistant bacteria into water, likely also constituting an important mechanism for the dissemination of antibiotic-resistant bacteria (ARBs) in natural bacterial communities. ARBs are becoming a major global public health problem [10,11], and the extensive use of antimicrobial treatments in humans and animals may increase resistant bacterial populations in the aquatic environment [12]. Most antibiotic resistance research has focused on isolates from clinical patients, but ARBs are widespread in the environment, including water and soil [11]. In 2013, Bassaseachic Falls National Park in Chihuahua presented diarrhea outbreaks among inhabitants of the park, primarily in children, probably caused by contaminated water consumption. The aim of the present study was to identify the multidrug-resistant enteric pathogenic and native bacteria present in the surface water of Bassaseachic Falls National Park in Chihuahua, Mexico as a possible source of water outbreaks.

## 2. Experimental Section

### 2.1. Sample Collection

**Figure 1:**
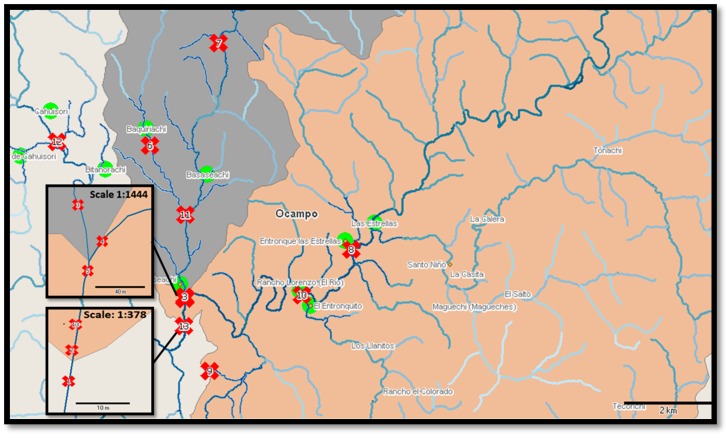
General scheme of Bassaseachic Falls National Park. The (**X**) symbols indicate the sampling sites: (●) Rural settlement within the studied area. Source: INEGI digital map, Cartographic Package 2010; Scale 1:64140. Samples 5 (28°10′47.45″N, 108°12′45.49″W) and 6 (28°12′40.80″N, 108°13′20.49″W) were lost during the summer, and sample 13 was lost during the spring (28°10′27.36″N, 108°12′44.97″W).

Thirteen reference locations situated near rural settlements, waterfall wells, and the belvedere used by inhabitants to collect water for drinking, cooking or washing purposes, were selected in the Bassaseachic Falls National Park, Ocampo Municipality in the state of Chihuahua, Mexico (28°07′59″N 108°15′00″W, Figure 1).

A total of 49 sampling points were selected from 13 different points described in Table 1, and each location was selected near principal rural settlements. Samples 1, 2, and 13 refer directly to Basaseachi Falls; in addition, two samples were collected from the visitors’ center (sample 9) and a small waterfall (sample 2) next to the Basaseachi waterfall. Four 1-L samples were collected at each location using 1.2-L polypropylene bottles previously sterilized under UV light for 3 h, according to Mexican sampling standards [13]). Each sample was collected approximately 30 cm below the water surface, and the bottles were open only under the water. Four liters of water from each sampling point was stored upright on ice in 1.2-L polypropylene bottles until further laboratory analysis. The seasons were defined as winter (December), spring (March), summer (June) and autumn (October). Two of the total water samples were lost as a result of the dry spell during the summer (samples 5 and 6), and one water sample was lost during the spring as a result of unsafe sampling conditions (sample 13). All collection sites were identified using GPS (Table 1). The presence of native and enteropathogenic bacteria was determined using microbiological and biochemical techniques for the identification of Gram-negative bacteria from all collected samples. 

### 2.2. Microbiological Analysis

#### 2.2.1. Total/Fecal Coliform Determination

Total and fecal coliform counts were determined using the multiple-tube fermentation technique and were reported as the most probable number (MPN) of organisms present per 100 mL of sample. The MPN analysis was performed within 24 h of sampling, according to the Mexican Standard [14]. Briefly, 10-, 1-, and 0.1-mL aliquots of the season/site samples were inoculated in triplicate into 10 mL of lactose broth (Merck, Naucalpan de Juárez, México) in an inverted Durham tube and incubated at 37 °C for 24 h; gas formation indicated a positive result. One milliliter of the gas-positive samples was inoculated into 10 mL of standard broth for total coliform counts and was incubated at 44 °C. Another milliliter of the gas-positive sample was inoculated in brilliant green broth for fecal coliform confirmation and was incubated at 37 °C. The negative samples were incubated for an additional 48 h as a confirmative test.

#### 2.2.2. Microbial Isolation

Water samples that exceeded the Mexican Standard (1000 MPN/100 mL) were analyzed using standard microbiological techniques for the enumeration of bacteria and the identification of pathogens. To isolate pure cultures, the freshwater samples were placed in pre-selection media, followed by selective media (Supplementary Material Figure S1). From the initial bacterial growth, pure culture isolates were separated and analyzed further through macroscopic and microscopic characterizations. Each isolate was characterized based on its colony macroscopic morphology (shape, size, type of edge, transmitted or reflected light) and Gram stain. For isolation, the method of Al-Bayatti *et al.* [15] was followed, with minor modifications. Briefly, to obtain pure cultures, 10 mL of freshwater sample was inoculated into 10 mL of BBL-buffered peptone water (Difco, supplied by Productos Químicos Viqmar, Tlalnepantla Estado de México, México), 2% sodium chloride broth (Sigma-Aldrich, St. Louis, MO, USA), tetrathionate broth (Difco) and selenite broth (Difco). The cultures were incubated overnight at 37 °C using an oven (model 314, Thermo Fisher Scientific, Waltham, MA, USA), and 10 mL of the freshwater sample was also inoculated into nutrient broth (MCD Lab, Tlalnepantla, Estado de México, México) and incubated overnight at 42 °C. The bacteria grown on selenite and tetrathionate broth as an original source were re-inoculated in Salmonella-Shigella (SS) to recover *Salmonella* and *Shigella* isolates (SS, Bioxon supplied by Productos Químicos Viqmar) and were incubated overnight at 37 °C. Bacteria grown in BBL-buffered peptone water and 2% NaCl broth were re-inoculated onto thiosulfate citrate bile salt sucrose agar (TCBS, BD Bioxon, Estado de México, México) to recover a variety of *Vibrio* spp. [16]. In addition to nutrient broth, the samples were re-inoculated onto nutrient agar to increase the range of the recovered Gram-negative bacteria and onto MacConkey agar to presumptively identify the *Escherichia coli* isolates*.* The purified colonies were subsequently cultured on MacConkey agar and TCBS for further analysis and stored at −20 °C in trypticase soy broth (Bioxon) containing 30% glycerol.

### 2.3. Biochemical Identification

Pure colonies were recovered from MacConkey and TCBS agar for biochemical identification using the AutoSCAN-4 system (DADE Behring MicroScan-Plus, Kennett Square, PA, USA) to identify fermentative and non-fermentative Gram-negative bacilli. The AutoScan ID panel was prepared according to the manufacturer’s instructions. Identification was based on the detection of pH changes determined via modified and chromogenic tests, substrate utilization, and growth in the presence of antimicrobial agents after 16–42 h of incubation at 35 °C. Rapid Combo panel NEG-44 was selected to identify Gram-negative bacteria based on conventional and chromogenic tests in 96-well microplates and on 27 dehydrated substrates. The results were analyzed using Labpro software to identify microorganisms and to generate an antibiotic resistance profile. Only Gram negative bacteria were further analyzed. Similarly, only one isolate from isolates showing similar macroscopic (colonial) and microscopic characteristic was selected.

### 2.4. Antibiotic Resistance Analyses

The antimicrobial susceptibility tests are miniaturizations of the broth dilution susceptibility test, using dehydrated samples. Pure colonies tested for biochemical identification were also tested to growth in the presence of antimicrobial agents for antibiotic resistance profiling using the AutoSCAN-4 system. The rapid combo panel NEG-44-tested antibiotics included: amikacin, ampicillin-sulbactam, ampicillin, aztreonam, cefazolin, cefepime, cefotetan, ceftazimide, ceftriaxone, cefuroxime, ciprofloxacin, gentamicin, imipenem, levofloxacin, meropenem, moxifloxacin, piperacillin-tazobactam, ticarcillin-clavulanic acid, tobramycin, and trimethoprim-sulfamethoxazole. The breakpoint combo panels used concentrations equivalent to the categorical breakpoints of the CLSI, and the minimum inhibitory concentrations (MICs) [17,18]. If two or more isolates from the same sampling point exhibited identical results according to the microscan biotype flinging number, then only one of the isolates was analyzed further. 

### 2.5. Statistical Analysis

For the MPN analysis, the data were normalized through the logarithmic transformation of the quantitative MPN results, and ANOVA with the general linear model was used to test for determinations of statistical significance. Comparisons among groups were obtained using Tukey test. Minitab statistical software was used for data analysis. For each season, the calculation of the geometric mean included all sites, whereas the calculation of the geometric mean for each site included all seasons, with confidence intervals of 95% (Minitab v17). 

## 3. Results 

### 3.1. Microbiological Analysis

**Figure 2:**
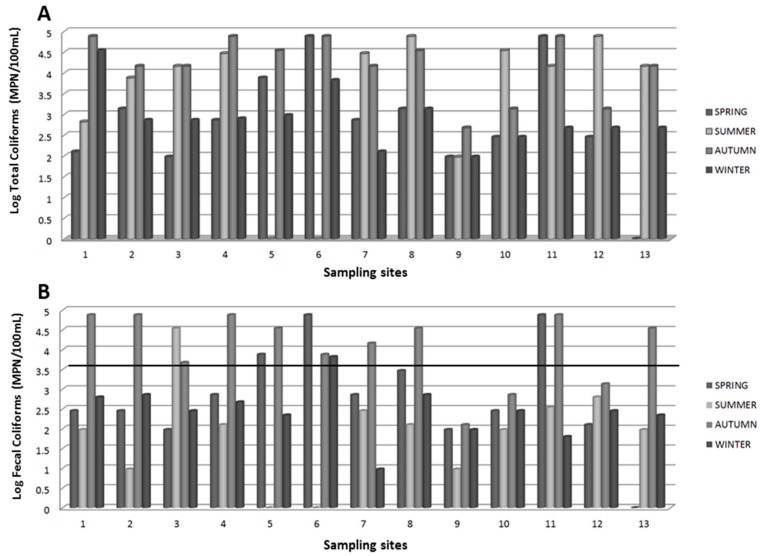
Log MPN counts of (**A**) total coliforms and (**B**) fecal coliforms per sampling site in the water samples collected from Bassaseachic Falls National Park, Ocampo, Chihuahua, México, during 2013. The black line across Figure 2B on 3.5 MPN indicates the [19].

The samples collected from Bassaseachic Falls National Park during 2013 were analyzed for total and fecal coliform enumeration. Results showed that calculated data were exceeding Mexican standards among total and fecal coliform counts. The total coliform counts from the sampling season and sampling sites are shown in Figure 2A. Similarly, the fecal coliform counts from the sampling season and sampling sites are shown in Figure 2B. The samples collected at site 9 showed the lowest total and fecal coliform counts compared with the other sample sites. 

In contrast, the samples collected from sites 5, 6, 8, and 11 exhibited the highest counts, where the total coliforms were higher than the fecal coliforms. Except for samples 9, 10 and 12, the collected water samples had greater than 1000 MPN/100 mL (3.5 MPN) fecal coliforms during the autumn and summer seasons.

**Figure 3:**
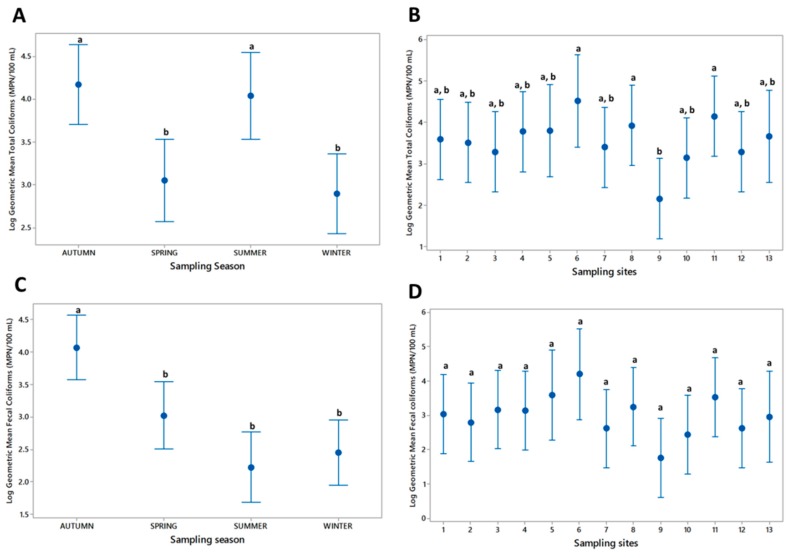
The geometric means considering all sites and all seasons in logarithmic scale and with confidence intervals of 95%. (**A**) Total coliforms per sampling season; (**B**) Total coliforms per sampling site; (**C**) Fecal coliforms per sampling season; (**D**) Fecal coliforms per sampling site. Bars showing different letter are significantly different (Tukey, *p* < 0.05).

The pooled MPN geometric mean was used to calculate the intervals, with a 95% confidence interval (Figure 3). The total coliform counts are shown in Figure 3A,B. ANOVA revealed significant differences (*p* < 0.001) between the total coliform counts from the autumn and summer (highest) and spring and summer (lowest) seasons. ANOVA also revealed significant differences between the total coliforms (*p* < 0.05) at the sampling sites. The results of Tukey test revealed that samples 6, 8, and 11 had the highest coliform counts and that sample 9 had the lowest count (Figure 3B).

The geometric mean of the fecal coliforms significantly varied among the seasons (*p* < 0.001), where most of the samples collected during autumn exhibited significant differences compared with the remaining samples collected during the 2013 seasons by Tukey test (*p* < 0.05) (Figure 3C). Tukey test also revealed no significant differences between the fecal coliform geometric means of the sampling sites from pooled seasons (*p* = 0.133; Figure 3D). 

### 3.2. Biochemical and Antibiotic Resistance Profiles

A total of 33 Gram negative isolates showing different macroscopic and microscopic characteristics were recovered from 23 river water samples. The 33 bacterial isolates were selected for biochemical identification and to determine their antibiotic resistance profile. Results revealed that all 33 belonged to *gammaproteobacteria*, whereas the AutoScan-4 analysis revealed 16 different Gram-negative genera and species. Isolates were identified as belonging to the *Enterobacteriaceae*, *Pasterurellaceae*, *Vibrionaceae*, and *Moraxellaceae* families (Table 2). 

The antibiotic susceptibility analysis to detect multidrug-resistant (MDR), extensively drug-resistant (XDR) or pandrug resistant (PDR) bacteria among enterobacteria isolates was performed using 21 different antibiotics. By a proposed definition, any strain presenting a resistance phenotype in ≥3 classes of antibiotics is an MDR bacterium [28]. The breakpoint combo panel for each isolate showing lower inhibition by each exposed antibiotic is listed in Supplementary Material Table S1 [17,18]. Antibiotic resistance results are listed in Table 3 and Supplementary Material Tables S1 and S2. Accordingly to the resistance phenotype definition, we isolated five MDR bacteria: 2/4 bacteria were identified as *K. oxytoca* (77704370 and 77714372), 2/7 bacteria were identified as *E. coli* (77115010 and 77113010), and 1/3 bacteria were identified as *E. cloacae* (77103173). The individual profiles of the single isolates showing resistance to at least one antibiotic revealed that *Hafnia alvei* exhibited intrinsic antibiotic resistance against either cefuroxime (biotype 43005103) or cefazolin (biotypes 43005103, 43005103 and 76103172). Similarly, *Aeromonas hydrophila* biotype 60010150 exhibited resistance against trimetroprim-sulfametoxazole and ampicillin-sulbactam, whereas *Tatumella* sp. (biotype 600000110) exhibited resistance against ampicillin or the ampicillin-sulbactam complex (Supplementary Table S2). 

**Figure 4:**
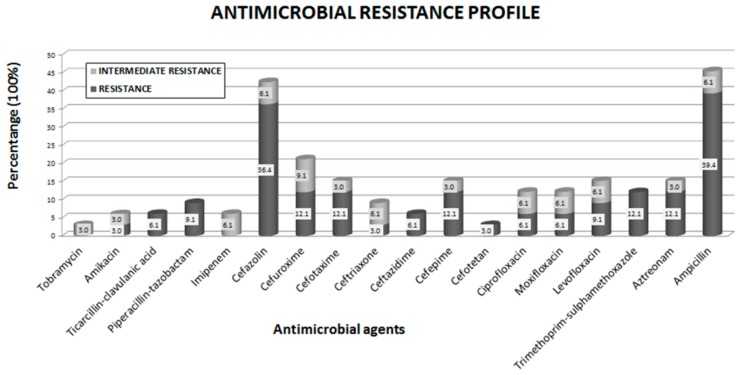
Antibiotic resistance profiles (%) among the isolates from samples collected during 2013 and sampling sites in the Bassaseachic Falls National park, Ocampo, Chihuahua, Mexico, using the AutoScan ID panel database.

An evaluation of the antibiotic resistance of the antibiotic tested revealed that all 33 isolates were susceptible to gentamicin, imipenem, meropenem and tobramycin. In addition, 3% to 6.1% of the isolates presented intermediate resistance to most other antimicrobials tested, including imipenem and tobramycin (Figure 4, Supplementary Table S2). 

Additionally, 39.4% of isolates were resistant to ampicillin; 36.4% of isolates were resistant to cefazoline; 21.2% of isolates were resistant to ampicillin-sulbactam; 12.1% of isolates were resistant to aztreonam, cefepime, cefotaxima, cefuroxima and trimethoprim-sulfamethoxazole; 9.1% of isolates were resistant to levofloxacin and piperacillin-tazobactam; and 6.1% of isolates were resistant to ceftazidime, ciprofloxacin, moxifloxacin and ticacillin-clavulanic acid. Only 3% of isolates were resistant to amikacin, cefotetan and ceftriaxone. In the present study, *Tatumella* sp*.* did not exhibit resistance to any antibiotic tested; however, this bacterium exhibited intermediate resistance to imipenem, similar to the *K. oxytoca* isolate. In the present study, we identified one *E. coli* isolate exhibiting intermediate resistance to tobramycin (Supplementary Table S2). The antibiotic resistance profiles comparing freshwater native and enterobacterial species/biotypes collected at Basaseachi river revealed intermediate resistance in 48% (16/33) of all isolates detected in the present study (Supplementary Table S2). 

## 4. Discussion

Basaseachi river and waterfalls is the second-highest waterfall (807 feet tall) in Mexico and is located in the Bassaseachic Falls National Park at Cañon Basaseachi in the Copper Canyon region of northwest Mexico, near Creel, Chihuahua. Diarrheic outbreaks have been observed among inhabitants of this area, whom lack to access to non-contaminated water and appropriate sewage disposal. Unfortunately, clinical official data are not available due to ill inhabitants’ poverty and deficiencies in sample analyses. Moreover, medical facilities are insufficient and conventional antibiotic treatments are not quite effective to control the diarrhea symptoms. As a result, most ill people with acute diarrhea must go to other closer hospitals to get proper medical attention, being more difficult to get clinical data of Basaseachi inhabitants. These diarrheic outbreaks have prompted to evaluate the water contamination, looking after responsible microorganisms that may be related to this outbreak event, and if those microorganisms are resistant to the recommended antibiotics for diarrhea medical control (project approved by the National Commission of Protected Natural Areas CONANP/DR03/08/PN01/PROCODES/1259/13). The aim of the present study was to identify the populations and antibiotic resistance profiles of coliforms present in the Basaseachi river and streams within the park to detect microbial contamination that may be resistant to conventional drug treatments. The samples were collected near rural settlements, including the waterfall well and the belvedere of Bassaseachic Falls National Park.

Mexican standard regulations established a limit of 1000 MPN/100 mL (3.5 MPN) fecal coliforms from sewage pollutants dumped in natural environments [19]. After analyzing the total coliform populations among the seasons, the highest population was detected during rainfall in the summer of 2013 (Figure 3A). For fecal coliform counts, autumn exhibited the highest value among the seasons of the year (Figure 3C). The results may indicate water movement during rain and higher temperatures (above 35 °C in daylight) during summer. In contrast, the lowest total coliform counts were observed in summer and winter as lower rain/humidity and temperatures (not higher than 20 °C) are present (Figure 3C). Davino *et al.* [29] reported similar results after analyzing the coliform counts in Jatiúca Beach, Brazil. These authors reported that the fecal coliform count was higher during the wet seasons (May, June, and July) than during the dry seasons (November, December, and January). 

Analysis of the data for total and fecal coliform enumerations at the sampling sites revealed that, except for sampling sites 9, 10, and 12, the collected samples possessed more than 1000 MPN/100 mL (3.5 MPN) fecal coliforms during the autumn and summer seasons (Figure 2A,B). Samples 6 and 8 were collected near the biggest rural settlements, and sample 11 was obtained from an oxidation lagoon that might be filtered into the Bassaseachic Falls National Park River. Sample 9, which exhibited the lowest coliform count, was collected from a rural settlement belonging to the park keepers and inhabited by approximately 20 people. This site was likely associated with lower sanitary discharges and fecal pollution (Figure 2A,B).

A total of 33 Gram negative isolates showing macroscopic and microscopic differences (*gamma-proteobacteria*) were selected to identify enterobacteria populations present in the Bassaseachic Falls National Park River. Biochemical identification analyses grouped them in 16 different genera and species from the *Enterobacteriaceae*, *Pasterurellaceae*, *Vibrionaceae,* and *Moraxellaceae* families (Table 2). Selected isolates identified in the present study are commonly reported in epidemic water outbreaks in developing countries [4,9,30], thereby explaining the diarrhea outbreaks observed among the Basaseachi waterfalls inhabitants, primarily the children. Gastrointestinal diseases were associated with most bacterial pathogens found in this study, and linked with wastewater contamination to well water (Table 2). The identified genera included *Escherichia*, a fecal indicator of polluted waters [31]. Furthermore, the presence of *Shigella* sp., a strictly human pathogen, may also be associated with human fecal pollution [32]. Bacterial pathogens are considered water pollutants (biological contaminants) due to *the* run-off from urban and agricultural areas, leakage from sewers and septic systems, and sewer overflows [33]. The presence of *Salmonella* sp. in food and water is considered a Public Health risk, since such as bacterium is not a common water inhabitant [34]. 

*Salmonella* is the most frequent causative agent of bacterial gastroenteritis [34,35]. It is difficult to isolate from environmental water samples; nevertheless, the presence of *Salmonella* represents a Public Health risk as the infective dose can be as low as 15 to 100 bacterial cells per milliliter [34]. Furthermore, chicken is a major vehicle for the transmission of this bacterium*,* which is considered a major problem in the poultry industry [35]. The chicken poultry breeding is a common practice within the Basaseachi river inhabitants. 

In addition, *Yersinia* has been associated with seafood contamination and water outbreaks, whereas *E. coli* O157:H7, frequently isolated from waters worldwide, has been identified in 2% of raw sludge [32]. Multidrug resistance patterns have been also detected in *E. coli* isolates isolated from river water in Osun State, Nigeria [36] and from the holy city of Mathura, India [37]. 

Nevertheless, seven of our enterobacterial isolates are rarely pathogens, but they were observed in nosocomial infections, mostly among immunosuppressed patients [21]. Diseases related with these less common enteropathogens include acute diarrheas, gastroenteritis, septicemia, meningitis, and wound infections by *A. hydrophila* [20,21]; diarrhea by *C. freundii* (after having or acquiring the ability to produce an enterotoxin) [21,22]; community-acquired and nosocomial infections such as urinary tract infections and bacteremia by *E. cloacae* [21]; acute diarrhea and Traveler’s diarrhea by *Hafnia alvei* [38]; nosocomial intestinal and urinal infections by *K. oxytoca* [21]; gastroenteritis by *Tatumella* sp. [24]; and gastritis, cellulitis, endocarditis, endophthalmitis by *A. lwoffii* [27]. The only isolated bacterium that has not been associated with water contamination, but by an infected animal direct contact is *P. multocida*. Nevertheless, in a 30-year study, the Detroit Medical Center observed this bacterium causing infections in 14 patients, half of which reported no contact with animals [26]. 

As stated before, *Klebsiella* members are opportunistic nosocomial pathogens; only *K. pneumoniae* and *K. oxytoca* are human pathogens. Hospital outbreaks frequently result from a new strain type of multidrug-resistant *Klebsiella* sp. Among the environmental water samples, the number of *Klebsiella* spp. was low (usually one to five colony-forming units [CFU]/250 mL), regardless of season. Podschun *et al.* [39] reported that among the *Klebsiella* sp. isolated, *K. pneumoniae* was the most common (52%), followed by *K. oxytoca* (27%). In the present study, the *K. oxytoca* population was higher (four isolates) compared with the *K. pneumoniae* population (one isolate). In addition, 50% of the *K. oxytoca* isolates (2/4) were identified as multidrug-resistant bacteria. 

As previously described, after two or more isolates from the same sampling point were identified as identical (same biotype flinging number), only one isolate was selected for further analyses. Seven of 33 isolates total were identified with the same biotype, thus suggesting they were similar strains; however, these strains were isolated from different sample points and did not present the same resistance profile [1,40].

*A. lwoffi*-like species are commonly isolated from pelletized food, and *A. hydrophila* has been associated with fish disease [20,41], whereas the *Tatumella* sp. [42] has been associated with soil inhabitants [43]. Similarly, *H. alvei* has been isolated from mammals, birds, reptiles, fish, soil, water, and sewage samples. These enteric bacteria are considered non-human-associated bacteria and rare opportunistic pathogens [38]. *A. lwoffii, A. hydrophila,* and *Tatumella* sp*.* are saprophyte bacteria. In the wild, these microbes are not considered human health risk bacteria [7,44]. 

In the present study, we evaluated the antibiotic susceptibility of 33 selected enterobacterial isolates from the collected freshwater samples to identify bacteria considered as Public Health threats by showing multidrug resistance patterns. According to the international standard definitions for any MDR microorganism [28], we isolated five MDR bacteria; two isolates were identified as *K. oxytoca*, two as *E. coli*, and one as *E. cloacae*. Nevertheless, same genera and species isolates were identified, representing the 50% (2/4) the MDR isolates identified as *K. oxytoca*, 28.57% (2/7), the MDR isolates identified as *E. coli* and 33.33% (1/3) the MDR isolate identified as *E. cloacae*. This pattern of resistance to several key antibiotics commonly used in therapeutic treatments is considered a public health threat [28]. Similarly, MDR isolates from the *Enterobacteriaceae* family have been identified in the Almendares River in Cuba [45]. As mentioned above, from 33 isolates, seven presented a similar biotype; however, identical isolates did not possess the same resistance profile, thus indicating a potential antibiotic resistance selection [1,40,44].

Active antibiotics can be discharged in considerable amounts in the forms of human waste. Native bacteria in natural environments can be exposed to these antibiotics, particularly those with higher persistence in the environment, such as fluoroquinolones and tetracyclines. Fluoroquinolones and tetracyclines are more stable and remain in the environment for longer periods of time, facilitating bacterial antibiotic resistance selection [46]. The increasing rate of antibiotic-resistant microorganisms is recognized as a serious ecological problem [47]. 

Overall, the results of the present study demonstrated that the antibiotic resistance patterns detected in the isolates collected from Bassaseachic Falls National Park, may be the reason for the ineffective control using antibiotics to control gastrointestinal infections, resulting in lower alternatives for therapeutic treatments. Some of the identified bacteria in the present study were coliforms/enteropathogenic species, thus indicating a human or animal waste source. Animal and human waste, mostly generated in rural locations, is discharged into water bodies, and play an important role in diarrhea outbreaks observed within inhabitants of this northwest Mexican area.

## 5. Conclusions

Surface water samples from Bassaseachic Falls National Park revealed the presence of bacterial pathogens that are considered public health risks. Five isolates showing multidrug (antibiotic) resistance (MDR) profiles from this natural water environment were identified. Overall, the microorganisms detected may cause future problems and represent a health threat to the people who live near this park. MDR agents constitute a major threat to Public Health and should receive more attention. The monitoring of antibiotics resistance genes and the presence of resistant bacteria in the environment are increasingly viewed as ecological problems for which further studies are needed to provide solutions that preserve seasonal water bodies, such as ponds, rivers, lakes, and waterfalls, and their productive ecosystems. 

## Figures and Tables

**Table 1:** Description of the freshwater sample sites at Bassaseachic Falls National Park during 2013.

Sample Points		Geographical Coordinates	
Sample	Sample Site	North (N)	West (W)
1	Basaseachi waterfall well	28°10′26.95″N	108°12′44.99″W
2	“La ventana” waterfall	28°10′27.17″N	108°12′44.88″W
3	“El Durazno” river	28°10′48.24″N	108°12′45.09″W
4	Basaseachi river	28°10′49.19″N	108°12′45.86″W
5	Y. Basaseachi junction of the river and Durazno river.	28°10′47.45″N	108°12′45.49″W
6	“Baquiriachi” stream	28°12′40.80″N	108°13′20.49″W
7	Basaseachi water supply stream	28°13′59.55″N	108°12′26.07″W
8	“Las Estrellas” stream	28°11′30.06″N	108°10′27.09″W
9	Visitors center of Bassaseachic Falls National Park	28°09′54.7″N	108°12′21.7″W
10	“Betorachi” stream	28°10′54.08″N	108°11′6.84″W
11	Creek next to the oxidation pond	28°11′50.25″N	108°12′47.88″W
12	“Cahuisori” stream	28°12′40.58″N	108°14′38.92″W
13	Belvedere of Basaseachi waterfall	28°10′27.36″N	108°12′44.97″W

**Table 2:** Identified bacterial isolates recovered from freshwater at Bassaseachic Falls National Park during 2013.

Family	Isolate Identification and References ^1^	Number of Isolates	Sampling Site	Sampling Season
*Enterobacteriaceae*	*Aeromonas hydrophila* (Chester) Stanier [20]	1	3	Autumn
	*Citrobacter freundii* Werkman & Gillen [21,22]	2	2,5	Summer–Winter
	*Enterobacter cloacae* Jordan/Hormaeche [21]	3	1,3,6	Autumn–Winter
	*Escherichia coli* Escherich [21]	7	1,6,11,13	Spring-Summer-Autumn-Winter
	*Hafnia alvei* Møller [22]	3	1,2,3	Summer-Autumn
	*Klebsiella oxytoca* Flügge/Lautrop [21]	4	1,8,10,11	Spring-Summer
	*Klebsiella pneumoniae* Uber [21]	1	11	Summer
	*Salmonella* spp. [23]	1	5	Autumn
	*Salmonella* *enterica* subsp. *enterica* serovar Paratyphi A (Ex Kauffmann & Edwards) Le Minor & Popoff [21,23]	2	1,12	Spring-Winter
	*Shigella* sp*.* Castellani & Chalmers [21]	1	13	Spring
	*Tatumella* sp*.* Hollis *et al.* [24]	1	13	Spring
	*Yersinia enterocolitica* (Schleifstein & Coleman) [25]	1	10	Spring
*Pasterurellaceae*	*Pasteurella multocida* Pasteur [26] ^2^	2	4,7	Spring
*Vibrionaceae*	*Vibrio cholerae* Pacini [21]	2	11,12	Summer-Autumn
	*Vibro parahemolyticus* Fujino *et al.*/Sakazaki *et al.* [21]	1	8.11	Summer
*Moraxellaceae*	*Acinetobacter lwoffii* Brisou & Prévot [27]	1	6	Winter

^1^ References of diarrhea (including diarrheic outbreaks) by the bacteria found in this study, linked to wastewater contamination to well water. ^2^ Infection is linked to an infected animal exposure, but in bacteremia cases, 50% of patients reported no contact with animals [26]

**Table 3:** Identification of enterobacteria demonstrating antibiotic resistance using the AutoScan ID panel database.^1^

Antimicrobial Category	Antimicrobial Agent	Antimicrobial Susceptibility	Species with Intrinsic Resistance to Antimicrobial Agents or Categories (51)a
Aminoglycosides	Gentamicin	S	All isolates
	Tobramycin	IR	*Escherichia coli* (1/7) (IR)
	Amikacin	R	*Escherichia coli* (1/7) *Klebsiella oxytoca* (1/4) (IR)
Antipseudomonal penicillin + β-lactamase inhibitors	Ticarcillin-clavulanic acid	R	*Escherichia coli* (1/7)
	Piperacillin-tazobactam (Pip/tazo)	R	*Enterobacter cloacae* (1/3) *Escherichia coli* (1/7) *Klebsiella oxytoca* (1/4)
Carbapenems	Imipenem	IR	*Klebsiella oxytoca* (1/4) (IR) *Tatumella* sp. (1/1) (IR)
	Meropenem	S	All isolates
Non-extended spectrum Cephalosporin: 1st and 2nd generation cephalosporins	Cefazolin	R	*Escherichia coli* (1/7) *Escherichia coli* (1/7) (IR) *Klebsiella oxytoca* (2/4) *Klebsiella oxytoca* (1/4) (IR) *Vibrio parahaemolyicus* (1/1) (Vibrionaceae)
	Cefuroxime	R	*Enterobacter cloacae* (1/2) *Escherichia coli* (1/7) *Klebsiella oxytoca* (1/4) *Klebsiella oxytoca* (1/4) (IR) *Vibrio parahaemolyicus* (1/1) (Vibrionaceae) (IR)
Extended-spectrum Cephalosporin: 3rd and 4th generation cephalosporins	Cefotaxime or ceftriaxone	R	*Aeromonas hydrophila* (1/1) (IR) *Citrobacter freundii* (1/2) (IR) *Enterobacter cloacae* (1/3) (IR) *Escherichia coli* (1/7) *Klebsiella oxytoca* (3/4) *Salmonella enterica* subsp*. enterica, serovar Paratyphi A* (1/2)
	Ceftazidime	R	*Citrobacter freundii* (1/2) *Enterobacter cloacae* (1/3) *Escherichia coli* (1/7)
	Cefepime	R	*Escherichia coli* (1/7) *Enterobacter cloacae* (1/3) *Klebsiella oxytoca* (1/4) *Klebsiella oxytoca* (1/4) (IR)
Cephamycins	Cefotetan	R	*Klebsiella oxytoca* (1/4)
Fluoroquinolones	Ciprofloxacin	R	*Escherichia coli* (2/7) *Escherichia coli* (1/7) (IR) *Klebsiella oxytoca* (1/4) (IR)
	Moxifloxacin	R	*Escherichia coli* (2/7) *Escherichia coli* (1/7) (IR) *Klebsiella oxytoca* (1/4) (IR)
	Levofloxacin	R	*Escherichia coli* (2/7) *Escherichia coli* (1/7) (IR) *Klebsiella oxytoca* (1/4) *S. enterica* subsp*. enterica,* serovar Paratyphi A (1/2) (IR)
Folate pathway inhibitors	Trimethoprim-sulfamethoxazole	R	*Aeromonas hydrophila* (1/1) *Escherichia coli* (3/7)
Monobactams	Aztreonam	R	*Citrobacter freundii* (1/2) (IR) *Enterobacter cloacae* (1/2) *Escherichia coli* (2/7) *Klebsiella oxytoca* (1/4)
Penicillin	Ampicillin	R	*Escherichia coli* (2/7) *S. enterica* subsp*. enterica,* serovar Paratyphi A (1/2) (IR) *Shigella* sp. (1/1) *Tatumella* sp. (1/1) *Vibrio cholerae* (2/2) (Vibrionaceae)
Penicillin + β-lactamase inhibitors	Ampicillin-sulbactam	R	*Aeromonas hydrophila* (1/1) *Enterobacter cloacae* (1/3) *Escherichia coli* (2/7) *Hafnia alvei* (2/3) (IR) *Klebsiella oxytoca* (1/4) *Shigella* sp*.* (1/1) *Tatumella* sp*.* (1/1)

^1^ Enterobacteria were isolated from samples collected during 2013 at Bassaseachic Falls National park, Ocampo, Chihuahua, Mexico. See information regarding the biotypes in Supplementary Table S2. Antimicrobial categories: R = resistant; S = susceptible; IR = Intermediate resistance.

## Supplementary Materials

The following are available online at www.mdpi.com/1660-4601/13/6/597/s1, Figure S1: Diagram of the microbiology analysis flux, Table S1: Interpretative breakpoints, Table S2: Native and enterobacterial species/biotypes isolated from freshwater samples collected from the Bassaseachic National Park, México.
