# Supplementary material for: Multidrug-Resistant Bacteria Isolated from Surface Water in Bassaseachic Falls National Park, Mexico
Source: Int J Environ Res Public Health. 2016 Jun 16;13(6):597. doi: 10.3390/ijerph13060597 (PMC4924054; doi:10.3390/ijerph13060597)
Supplement: Supplementary file 1 [file ijerph-13-00597-s001.pdf]

Supplementary Materials: Multidrug-Resistant Bacteria Isolated from Surface Water in Bassaseachic Falls National Park, Mexico

Ma. Carmen E. Delgado-Gardea, Patricia Tamez-Guerra, Ricardo Gomez-Flores, Francisco Javier Zavala-Díaz de la Serna, Gilberto Erosa-de la Vega, Guadalupe Virginia Nevárez-Moorillón, María Concepción Pérez-Recoder, Blanca E. Sánchez-Ramírez, María del Carmen González-Horta and Rocío Infante-Ramírez

Sample:	Sample size:	Inoculated and incubated overnight at:	To recovered:	Re-Inoculated and incubated overnight at:	Analysis of macroscopic and microscopic morphology:	To obtained purified colonies previous medium were re-inoculated in:
		Selenite broth (37°C)	*Salmonella* & *Shigella* strains	SS Agar (37°C)		MacConkey Agar (37°C)
		Tetrathionate broth (37°C)	*Salmonella* & *Shigella* strains	SS Agar (37°C)	• Macroscopic: Shape, size, type of edge, transmitted or reflected light	MacConkey Agar (37°C)
Raw water 1 L	10 mL of raw water	2% sodium chloride broth (37°C)	*Vibrio* sp.	TCBS Agar (37°C)		TCBS Agar (37°C)
		BBL buffered peptone water (37°C)	*Vibrio* sp.	TCBS Agar (37°C)	• Microscopic: Gram Stain	TCBS Agar (37°C)
		Nutrient broth (42°C)	Pathogen enterobacteria (*Escherichia coli* sp.)	MacConkey Agar (37°C)		MacConkey Agar (37°C)

Figure S1. Diagram of the microbiology analysis flux. Only samples that exceeded Mexican Standard (1000 MPN/100 mL) were processed as indicate. Isolates were preserve in trypticase soy broth with glycerol 30% vol:vol at −20 °C storage.

Table S1. Interpretative breakpoints.

Antimicrobial Agents	Bacteria	Susceptible (μg/mL)	Intermediate (μg/mL)	Resistance (μg/mL)
**Amikacin**	Enterobacteriaceae & *Vibrio cholerae*	≤16	32	≥64
**Ampicillin**		≤8	16	≥32
**Ampicillin-sulbactam**	Enterobacteriaceae & *Acinetobacter* spp.	≤8/4	16/8	≥32/16
**Aztreonam**	Enterobacteriaceae	≤8	16	≥32
**Cefazolin**		≤8	16	≥32
**Cefepime**		≤8	16	≥32
**Cefotaxime**		≤8	16–32	≥64
**Cefotetan**		≤16	32	≥64
**Ceftazidime**		≤8	16	≥32
**Ceftriaxione**		≤8	16–32	≥64
**Cefuroxime**		≤4	8–16	≥32
**Ciprofloxacin**		≤1	2	≥4
**Gentamicin**		≤4	8	≥16
**Levofloxacin**		≤2	4	≥8
**Imipenem**		≤4	8	≥16
**Meropenem**		≤4	8	≥16
**Moxifloxacin**		≤2	4	≥8
**Piperacillin-tazobactam**	Enterobacteriaceae	≤64/4	32/4	≥128/4
	Pseudomonas aeruginosa & Gram negative bacteria	≤16/4	64/4	≥128/4
**Ticarcillin-clavulanic acid**		≤64/2	32/2	≥128/2
**Tobramycin**	*Pseudomonas aeruginosa* & Gram negative bacteria	≤16/2	64/2	≥128/2
**Trimetroprim-sulphamethoxazole**		≤4	8	≥16

Table S2. Native and enterobacterial species/biotypes isolated from freshwater samples collected from the Bassaseachic National Park, México

Antimicrobial Category	Antibiotics Tested	Sample Number																							
		1	3	4	5	8	9	10	11	12	16 *	17 *	18	19	20	21 *	22 *	23	24	26	28	29	30	31	33 *
Aminoglycosides	Tobramycin													I											
	Amikacin											R					I								
	Gentamicin																								
Carbapenems	Imipenem					I						I					I								
	Meropenem												R	R											
Non-extended spectrum Cephalosporin: 1st and 2nd generation	Cefazolin		R	R	R		R	R	R	R	I	R				R	R	I					R		R
	Cefuroxime		R		I							R				I	R						I		R
Extended-spectrum Cephalosporin: 3rd and 4th generation	Cefotaxime											R				R	R			R					I
	Ceftriaxione	I						I																	
	Ceftazidime											R													R
	Cefepime						R					R				I	R								R
Cephamycins	Cefotetan																R								
Fluoroquinolones	Ciprofloxacin											I	R	R			I								
	Moxifloxacin											I					I								
	Levofloxacin											I	R	R			R								
Sulfonamides	Trimetroprim-sulphamethoxazole	R										R	R	R											
Monobactams	Aztreonam										R	R					R								R
Penicillin	Ampicillin					R			I	R	R	R			R	R	R	R	R		R	R		R	R
Penicillin combinations	Ampicillin-sulbactam	R				R					R	R					R				R				R
	Piperacillin-tazobactam											R					R								
	Ticarcillin-clavulanic acid											R					R								R

Antimicrobial categories: R = resistant; I = Intermediate resistance.

	Biotype	Genera	Species		Biotype	Genera	Species		Biotype	Genera	Species		Biotype	Genera	Species
1	60010150	*Aeromonas*	*hydrophila*	10	77520002	*Citrobacter*	*freundii*	19	77115012	*Escherichia*	*coli*	28	40010010	*Shigella*	sp.
2	20424	*Acinetobacter*	*iwoffi*	11	76103172	*Enterobacter*	*cloacae*	20	77704370	*Klebsiella*	*pneumoniae*	29	60010101	*Vibrio*	*cholerae*
3	43005103	*Hafnia*	*alvei*	12	77103173	*Enterobacter*	*cloacae*	21 *	77714370	*Klebsiella*	*oxytoca*	30	41005007	*Vibrio*	*parahaemolyicus*
4	43005103	*Halnia*	*alvei*	13	77113010	*Escherichia*	*coli*	22 *	77714376	*Klebsiella*	*oxytoca*	31	40005007	*Vibrio*	*choleare*
5	43005103	*Hafnia*	*alvei*	14	53111010	*Escherichia*	*coli*	23	77714372	*Klebsiella*	*oxytoca*	32	30000002	*Yersinia*	*enterocolítica*
6	60010001	*Pasteurella*	*multocida*	15	53317010	*Escherichia*	*coli*	24	77714370	*Klebsiella*	*oxytoca*	33 *	76103176	*Enterobacter*	*cloacae*
7	60010001	*Pasteurella*	*multocida*	16 *	77113010	*Escherichia*	*coli*	25	53101002	*Salmonella*	*paratyphi A*				
8	60000110	*Tatumella*	sp.	17 *	77115010	*Escherichia*	*coli*	26	73101006	*Salmonella*	*paratyphi A*				
9	77100002	*Citrobacter*	*freundii*	18	77115010	*Escherichia*	*coli*	27	53505041	*Salmonella*	sp.				

* = Multidrug-resistance bacteria.
